# Gut dysbiosis and neuroimmune responses to brain infection with Theiler’s murine encephalomyelitis virus

**DOI:** 10.1038/srep44377

**Published:** 2017-03-14

**Authors:** F. J. Carrillo-Salinas, L. Mestre, M. Mecha, A. Feliú, R. del Campo, N. Villarrubia, C. Espejo, X. Montalbán, J. C. Álvarez-Cermeño, L. M. Villar, C. Guaza

**Affiliations:** 1 Grupo de Neuroinmunología, Departamento de Neurobiología Funcional y de Sistemas, Instituto Cajal, CSIC, Madrid, Spain; 2 Red Española de Esclerosis Múltiple (REEM), Spain; 3 Servicio de Microbiología, Hospital Universitario Ramón y Cajal de Investigación Sanitaria (IRYCIS), Madrid, and Red Española de Investigación en Patología Infecciosa (REIPI), Sevilla, Spain; 4 Immunology Department, Hospital Universitario Ramón y Cajal de Investigación Sanitaria (IRYCIS), Madrid, Spain; 5 Servei de Neurología-Neuroimmunología, Centre d’Esclerosi Múltiple de Catalunya, Valld’Hebron Institut de Recerca, Hospital Universitari Valld’Hebron, 08035 Barcelona, Spain; 6 Universitat Autònoma de Barcelona, 08193 Bellaterra (Cerdanyola del Vallès), Spain; 7 Multiple Sclerosis Unit, Neurology Department, University of Alcalá, Madrid, Spain.

## Abstract

Recent studies have begun to point out the contribution of microbiota to multiple sclerosis (MS) pathogenesis. Theiler’s murine encephalomyelitis virus induced demyelinating disease (TMEV-IDD) is a model of progressive MS. Here, we first analyze the effect of intracerebral infection with TMEV on commensal microbiota and secondly, whether the early microbiota depletion influences the immune responses to TMEV on the acute phase (14 dpi) and its impact on the chronic phase (85 dpi). The intracranial inoculation of TMEV was associated with a moderate dysbiosis. The oral administration of antibiotics (ABX) of broad spectrum modified neuroimmune responses to TMEV dampening brain CD4^+^ and CD8^+^ T infiltration during the acute phase. The expression of cytokines, chemokines and VP2 capsid protein was enhanced and accompanied by clusters of activated microglia disseminated throughout the brain. Furthermore, ABX treated mice displayed lower levels of CD4^+^ and CD8^+^T cells in cervical and mesenteric lymph nodes. Increased mortality to TMEV was observed after ABX cessation at day 28pi. On the chronic phase, mice that survived after ABX withdrawal and recovered microbiota diversity showed subtle changes in brain cell infiltrates, microglia and gene expression of cytokines. Accordingly, the surviving mice of the group ABX-TMEV displayed similar disease severity than TMEV mice.

Multiple sclerosis (MS) is a chronic inflammatory demyelinating disease of the CNS with a quite varied clinical presentation that is coupled to heterogeneous histopathological features^1^. The inflammatory demyelination associated with MS is accompanied by neuronal and axonal degeneration, which leads to permanent neurological impairment^2^,^3^. Although, the precise aetiology of MS remains unclear, several factors have been linked to susceptibility to the disease, including genetic predisposition, autoimmunity and environmental factors^4^.

Alterations in the balance of the gut microbiome have been associated with detrimental or protective effects in experimental autoimmune diseases. Accumulating evidence supports the role of gut microbiota in guiding the maturation and functionality of the host’s immune system^5^. Gut microbiota can provide signals that fine tune the organism’s immune status, driving either immunoregulatory or effector activity^6^,^7^,^8^. Thus, commensal microbes fulfil a critical role in peripheral immune homeostasis and alterations to the commensal populations could affect the T regulatory cell (Treg)/Th17 equilibrium in the gut^9^. In addition, the resident microbes in the gut can influence immune responses distant from the mucosal surfaces^8^. Immunological dysregulation causes numerous human disorders, such as autoimmune diseases, and indeed, the commensal microbiota affects the auto-reactivity of peripheral immune cells to CNS self-antigens^10^. In this context, great interest has been generated regarding the contribution of microbiota to MS pathogenesis^6^. A larger proportion of MS patients develop antibodies against certain gastrointestinal antigens than healthy people, which would indicate alterations to the gut microbiota and in immune status associated with MS^11^. In the spontaneous experimental autoimmune encephalomyelitis (EAE) model of MS, germ-free (GF) status confers complete protection as a result of the attenuated Th-17 and B cell responses^12^. Moreover, in the induced models of EAE, GF mice developed a milder clinical course of EAE than conventionally colonized ones. GF immunized mice produce lower levels of IFN-γ and IL-17A although they generate more CD4^+^CD25^+^FoxP3^+^ Tregs^13^. Similarly, the treatment with antibiotics modulates the gut microbiota and attenuates EAE progression by inducing T, B and iNK Treg responses^6^,^14^.

Although one of the more established functions of the gut microbiota is to constitute a barrier that prevents the entry of external pathogens, some viral infections have been known to use the microbiota to their advantage^15^. Thus, successful transmission of a retrovirus (mouse mammary tumor virus, MMTV) depends on the commensal microbiota^16^ and poliovirus uses the gut microbiota to promote infection, thereby provoking a more severe clinical course^17^. Theiler’s murine encephalomyelitis virus (TMEV) is a mouse enteric pathogen that belongs to the single-stranded RNA picornaviruses. Experimentally demyelinating disease in susceptible mouse strains is induced by intracerebral infection with TMEV that produces a biphasic disease. During the first phase (the acute phase) the virus infects neurons and causes a mild and transient encephalomyelitis followed by a second phase (the chronic phase), during which the virus infects glial cells, primarily microglia, and macrophages of the spinal cord white matter. During this chronic progressive-induced demyelinating disease (TMEV-IDD) most of the lesions occur in the spinal cord associated with persistent viral infection in SJL/J mice^18^. TMEV-IDD serves as a relevant model of MS and indeed, the neurological dysfunction associated with the progressive axonal loss is similar to than seen in progressive forms of MS^19^,^20^. TMEV-IDD provides an unique example of how a pathogen inoculated in the brain causes inflammation, demyelination, autoimmunity, and neuronal damage resembling the pathological features of MS. Although a causative pathogen of MS has not yet been definitively identified, an infectious aetiology for this disease is still a current hypothesis^21^.

The administration of oral ABX that target a broad spectrum of bacteria has provided a complementary approach to study host-microbe interactions. Following ABX treatment, the density and composition of the intestinal microbiota is diminished dramatically in both humans and animals^22^. However, whether commensal gut bacteria can modify the early acute responses to TMEV as well as the ensuing clinical course and severity remains unknown. Hence, we set out to examine: (i) whether intracranial TMEV infection alters the commensal microbiota; (ii) whether altering the microbial load through oral administration of broad-spectrum ABX affects the immune responses to brain infection with TMEV during the acute phase of TMEV-IDD and (iii) the impact of early ABX treatment on the outcome and severity of the disease at chronic stages.

## Results

### CNS infection with TMEV is associated with changes in the commensal microbiota

**Figure 1:**
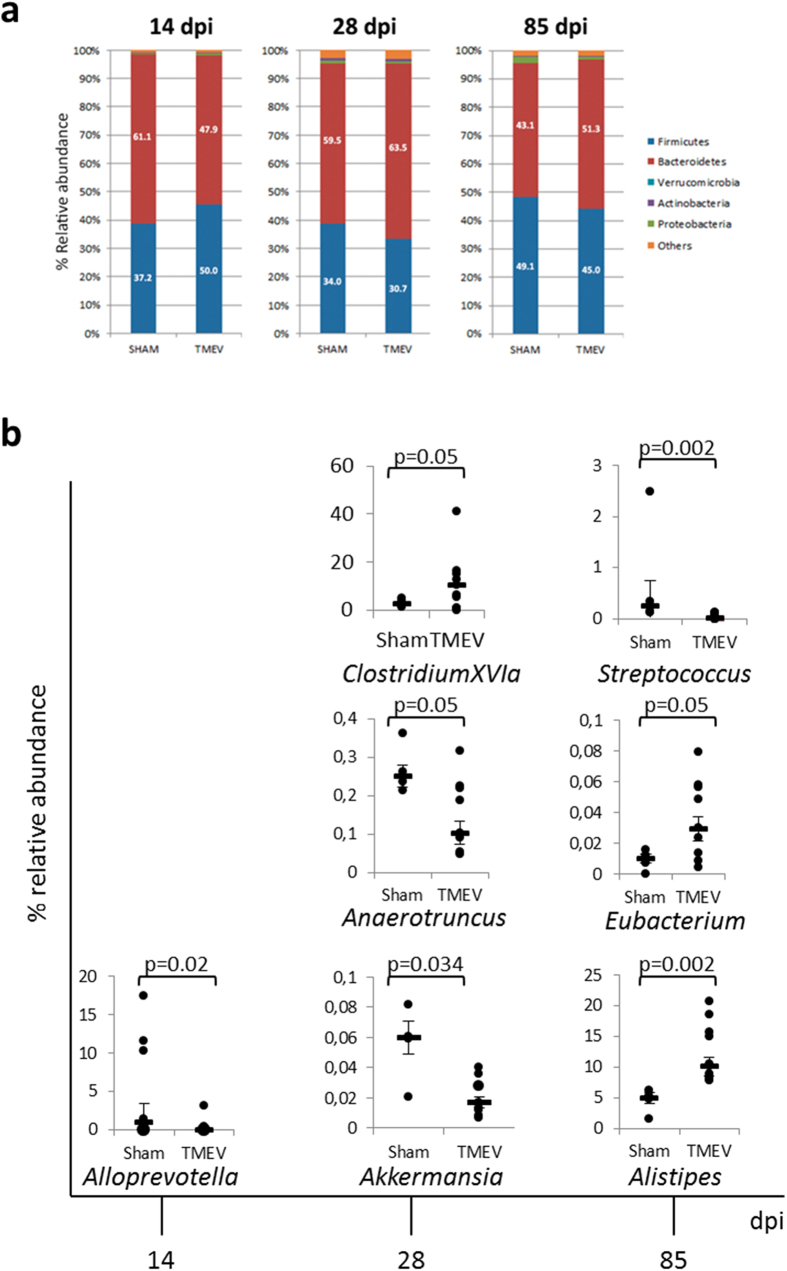
Theiler’s virus induces changes in the gut microbiota of susceptible SJL/J mice. Fecal pellets were collected at different times after TMEV infection (14, 28 and 85 dpi) (**a**) Stacked bar charts representing relative abundance of major phyla in the fecal microbiota of sham and TMEV-infected mice at 14, 28 or 85 dpi. (**b**) Plots of relative abundance of genera significantly different from fecal pellets of each mouse at 14, 28 or 85 dpi. Data are expressed as median of relative abundance ± SEM. Repeated measures ANOVA followed the post-hoc Bonferroni correction to compare the different sets in each time were analyzed. Sham (n = 4); TMEV (n = 9).

To date, the effects of brain infection with TMEV on the commensal gut microbiota have yet to be studied. Our results show that intracranial inoculation of TMEV was associated with alterations in the microbiota of SJL/J mice causing a moderate dysbiosis. During the acute phase at 14 days post infection (dpi) we observed an increase of the *Firmicutes* phylum that was further partially equilibrated at 28 dpi and at chronic stages, 85 dpi (Fig. 1a). At the genus level (Fig. 1b), TMEV-mice show a significant reduction of the relative abundance of *Alloprevotela* (phylum *Bacteroidetes*) at 14 dpi and a reduction of *Akkermansia* (phylum *Verrucomicrobia*) and *Anaerotruncus* (phylum *Firmicutes*) while *Clostridium XIVa* (phylum *Firmicutes*) increases at 28 dpi compared to sham mice. Indeed, at the chronic phase, the relative abundance of *Streptococcus* (phylum *Firmicutes*) was reduced in TMEV-mice, while *Alistipes* (phylum *Bacteroidetes*) and *Eubacterium* (phylum *Firmicutes*) were increased compared to sham (Fig. 1b).

Gut microbiota changes not only by environment but also throughout lifespan then, we studied the microbiota composition along time (14, 28 and 85 dpi) in sham and TMEV-mice and compared the alterations of relative abundance of microbiota between both groups as Table 1 summarized. Overall data indicate that the cerebral infection with TMEV induces subtle but significant changes in the gut microbiota at the time points analyzed.

### Oral treatment with ABX on the acute phase of TMEV-IDD affects the commensal microbiota on the course of the disease

**Figure 2:**
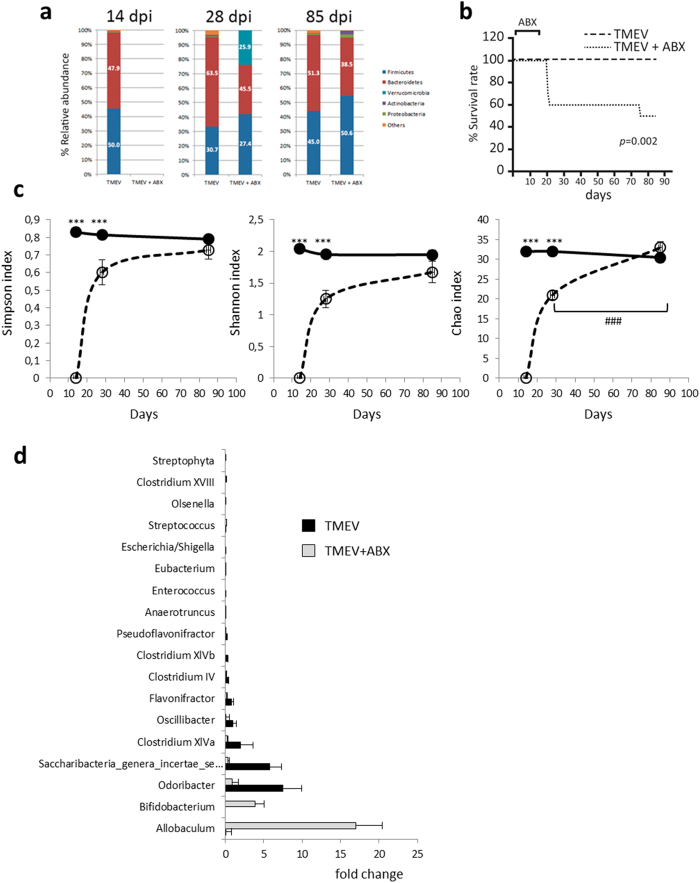
ABX treatment modifies the microbiota of TMEV-infected mice. Fecal pellets were collected at the end of ABX treatment (day 14), two weeks later (day 28) and in the chronic autoimmune phase of the disease model (evidence of clinical symptoms: day 85). (**a**) Stacked bar charts of the relative abundance of major phyla the three time points in the microbiota of TMEV-infected mice treated with ABX or vehicle. (**b**) A Kaplan-Meier log Rank statistical analysis was used to evaluate animal’s survival rate (**c**) Graphics of the estimated microbial diversity (Shannon and Simpson) and richness (Chao1) indexes of both TMEV and TMEV-ABX mice at 14, 28 and 85 dpi. (**d**) Plots of relative abundance of microbiota genera showing significant differences at 85 dpi. Data expressed as median ± SEM. Repeated measures ANOVA followed the post-hoc Bonferroni correction to compare the different sets in each time were used. When necessary, non-parametric Kruskal-Wallis test were used to assess the data. Statistics: ^***^p < 0.01 vs. TMEV; ^###^p < 0.001 *vs*. TMEV-ABX (28 dpi). TMEV (n = 9), TMEV-ABX (n = 10).

The influence of commensal microbiota on the host’s defense against TMEV has not been studied previously. We investigated the effects of oral ABX during the early acute phase of TMEV-IDD on subsequent commensal microbiota at 28 and 85 dpi. For this, mice orally received a combination of antibiotics (ABX: ampicillin, metronidazole, neomycin sulfate and vancomycin) for four-weeks, 2 weeks before TMEV infection and two weeks pi. This combination of ABX significantly altered the density and diversity of the gut microbiota in SJL/J mice^6^, a strain of mice susceptible to TMEV-IDD. Severe dysbiosis also occurred in C57BL/6 mice when this mix of ABX was administered for four weeks^23^ depleting the intestinal microbes of mice. Here, oral ABX depleted the intestinal microbiota and two weeks after ABX cessation recolonization was observed (Fig. 2a). However, this recolonization was incomplete at 28 dpi as TMEV-ABX mice have significantly more *Verrucomicrobia* and fewer *Bacteroidetes* members compared to TMEV (Fig. 2a). Strikingly, we observed a significant higher mortality at 21–22 dpi in TMEV-ABX mice as evidenced in the survival curve (Fig. 2b). Identical ABX treatment did not induce mortality in sham mice (data not shown). On the chronic phase (85 dpi) the diversity index (Simpson and Shannon) and the richness index Chao1 didn’t show significant differences between TMEV and TMEV-ABX mice (Fig. 2c). Therefore, the microbiota profile at 28 dpi was not maintained during the chronic phase of the disease, at least until 85 dpi, suggesting that there is a time dependent recovery of the gut microbes diversity after ABX withdrawal. Despite the recovery of microbiota diversity, TMEV-ABX mice show significant variations in the abundance of bacterial population at genus level within each of the main phyla (Fig. 2d). Accordingly, at 85 dpi early ABX increased the relative abundance of *Allobaculum* (phylum *Firmicutes*), and *Bifidobacterium* (phylum *Actinobacteria*) while decreased *Odoribacter* (phylum *Bacteroidetes*); *Olsenella* (phylum *Actinobacteria*), *Escherichia_Shigella* (phylum *Proteobacteria*); *Anaerotruncus, Clostridium IV, Clostridium XIVa, Clostridium XIVb, Clostridium XVIII, Enterococcus, Eubacterium, Flavonifractor, Oscillibacter, Pseudoflavonifractor, Saccharibacteria, Streptophyta, Streptococcus* (phylum *Firmicutes*).

### Intracerebral inoculation of TMEV increases intestinal permeability and the number of CD4^+^ T cells in the lamina propria of ileum during the acute phase

**Figure 3:**
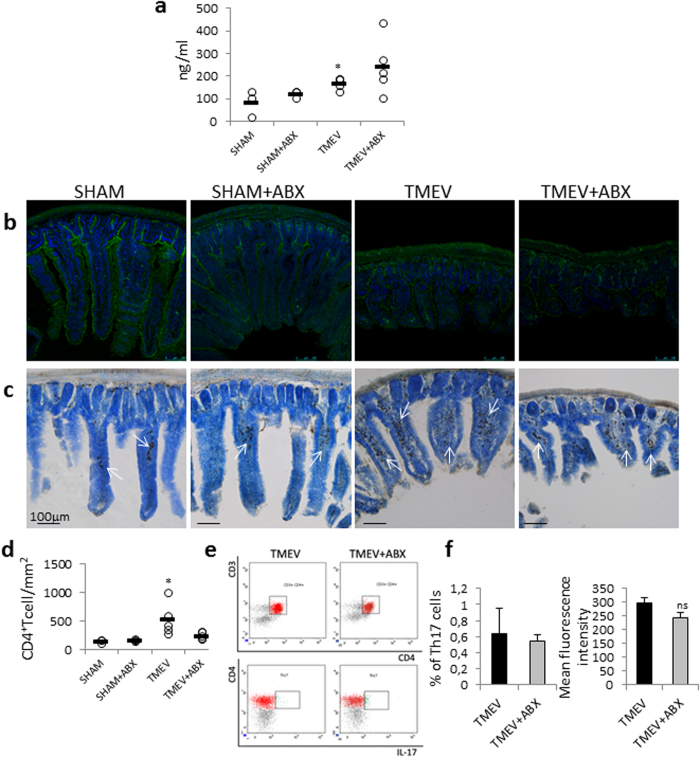
Intestinal permeability and CD4 T cells in the lamina propria were increased in TMEV-infected mice. (**a**) Mice were gavaged with a sodium fluorescein solution (100 μg/ml; Na-F, MW 376 Da) and one hour later the marker’s concentration in blood plasma were measured by the experimentalist using a spectrophotofluorometer. (**b**) Representative images of transversal ileum sections (30 μm) stained with ZO-1 for labelling tight junctions or with anti-CD4 (**c**) Arrows point to CD4^+^ T cells. Scale bar: 100 μm. (**d**) Quantification of number of CD4^+^ T cells/mm^2^ was performed on 6 sections for each animal. (**e**) Representative flow cytometry plots show CD3^+^CD4^+^ T cells expressing IL-17. (**f**) Quantification of the percentage of IL17-expressing CD3^+^CD4^+^ T cells in the Peyer’s patches of small intestine together with the mean fluorescence intensity in these cells. Data are expressed as mean ± SEM. Non-parametric Kruskal-Wallis test or Mann-Whitney U test were used to asses the significance of the data. ^*^p < 0.05 vs. sham. (sham, n = 3; sham-ABX, n = 5; TMEV, n = 5; TMEV-ABX, n = 5).

Next, we investigated whether brain TMEV infection could be affecting the intestinal mucosal barrier during the acute phase at day 14 pi. Then, to quantify whether intestinal permeability is altered in TMEV-mice or if the ABX treatment alone disrupts intestinal permeability we used the marker molecule Na-F by oral gavage (Fig. 3a). The concentration of the marker in blood was augmented in TMEV- mice indicating that the brain inoculation of the virus increased intestinal permeability when compared to sham mice (p < 0.05). Oral ABX did not modify permeability in sham mice. Although TMEV-mice show a tendency to increase permeability following ABX, this increase did not reach statistical significance. Next, we examined the expression of ZO-1, by immunofluorescence, in the ileum of sham and TMEV-mice subjected or not to ABX. Brain infection with TMEV affected the expression of ZO-1 showing a retraction on the villi compared to sham animals while ileum sections from TMEV-ABX mice show similar confocal images than those observed in sections from TMEV-mice (Fig. 3b). We also analyzed the number of CD4^+^ T cells in the lamina propria of ileum (Fig. 3c). Results show that brain TMEV inoculation was associated with an increase in the number of CD4^+^ T cells in the lamina propria without significant changes in TMEV–ABX mice (Fig. 3d). In addition, flow cytometry analysis showed that oral ABX did not modify Th-17 cells within the Pleyer’s patches (PP) compared to TMEV-mice (Fig. 3e,f).

### Oral ABX treatment enhances microglial activation in response to TMEV infection on the acute phase

**Figure 4:**
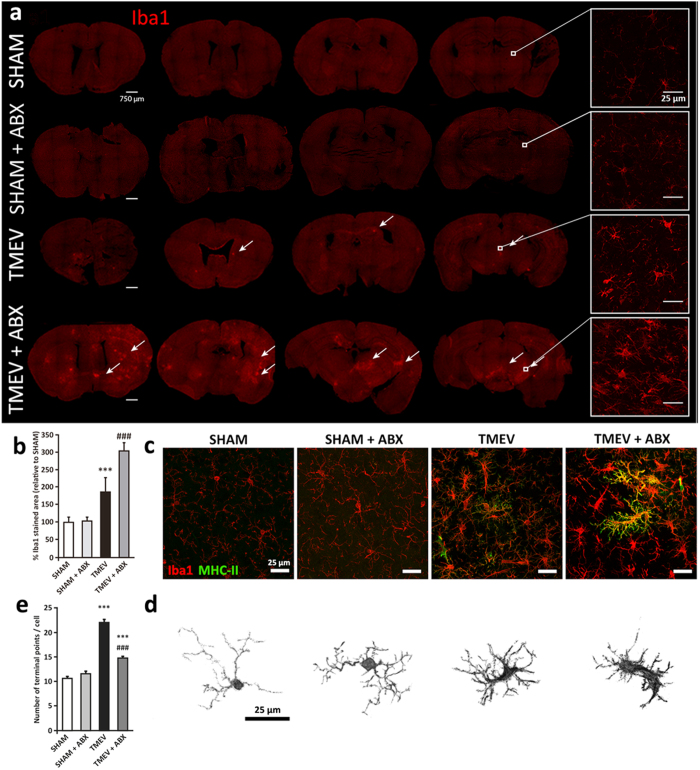
The depletion of microbiota by ABX treatment increases microglial reactivity in the brain of TMEV infected mice on the acute phase. (**a**) Immunofluorescence staining and laser scanning confocal microcopy was performed on representative brain coronal sections (30 μm) at different rostrocaudal levels from each group for the microglial marker Iba1. Scale bar 750 μm. Arrows point to Iba1 positive cluster of cells. Boxes show higher magnification image of Iba1 stained cells. Scale bar 25 μm. (**b**) Quantification of percentage of area occupied by Iba1 stained referred to sham mice. Two fields of cerebral cortex and striatum per section were measured from at least 6 sections for each animal, n = 4 animals/group. Non-parametric Kruskal-Wallis tests were performed: ^***^p < 0.001 vs sham; ^###^p < 0.001 vs TMEV. (**c**) Representative confocal fluorescent immunostaining of Iba1 (red) and MHC-II (green) in coronal brain sections (30 μm) from each experimental group. Scale bar 25 μm. (**d**) Representative isolated microglial cells images (**e**) quantification of endpoints per microglial cell obtained by Fiji software. Results are presented as mean ± SEM from more than 500 cells/animal from at least 6 sections for each animal. (n = 4 animals/group). Non-parametric Kruskal-Wallis tests were performed: ^***^p < 0.001 vs sham; ^###^p < 0.001 vs TMEV.

Intracerebral infection of SJL/J mice with TMEV provokes rapid multifocal inflammation in the CNS that involves resident microglia and cellular immune infiltrates. Microglia provide the first line of defense against TMEV infection by recognizing the virus through innate immune receptors^24^, although they may also be activated by recognition of damaged and dying cells during TMEV infection. We first evaluated microglial responses on day 14 pi through Iba1 immunostaining of the brain areas close to the virus injection in sham, sham-ABX, TMEV and TMEV-ABX mice, assessing the strength of microglia responses along the rostrocaudal brain axis (Fig. 4a). Oral ABX did not modify microglia morphology in sham mice nor Iba1 stained area (Fig. 4a–e). As expected, there were clear differences between sham and TMEV-mice, although the most significant changes corresponded to Iba1 staining that increased in brain sections from TMEV-ABX mice. Consequently, ABX administration enhanced microglial activation with clusters of activated cells more extensively disseminated across the cerebral cortex, hippocampus, striatum and brainstem than those observed in TMEV-mice that displayed more restricted Iba1 staining (Fig. 4a). Data quantification confirmed the increased microglial clusters in the brain of TMEV-ABX mice (p < 0.01; Fig. 4b). In an attempt to study the phenotype of microglia, Iba-1 co-staining with MHC class II and the cell morphology were analyzed. MHC class II antigens were overexpressed by microglia in TMEV-ABX mice compared with TMEV-mice (Fig. 4c). Moreover, TMEV-ABX mice showed reactive ameboid microglia with big soma and less terminal points compared to TMEV-mice. Sham mice, with and without ABX treatment, showed a resting microglia morphology (Fig. 4d,e).

### Oral ABX administration reduces CNS leukocyte infiltrates and modifies cytokines and chemokines expression in the cerebral cortex of TMEV-mice on the acute phase

**Figure 5:**
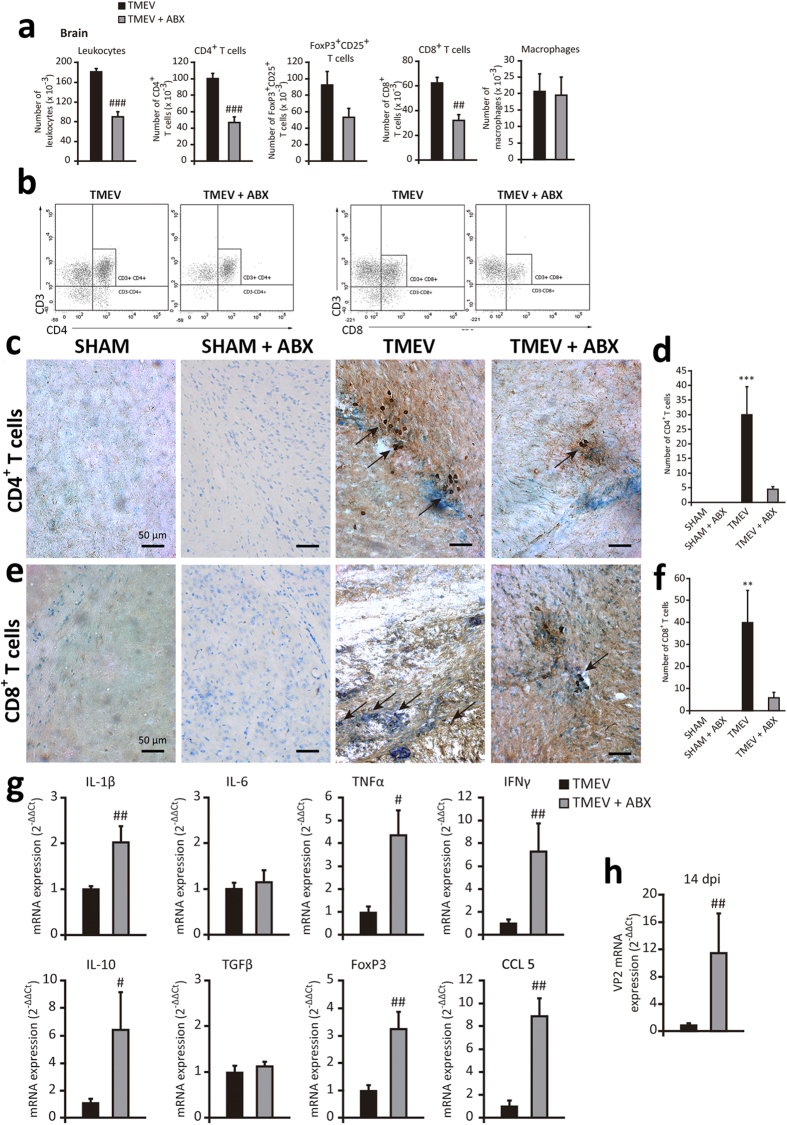
ABX treatment affects cell infiltrates into the brain parenchyma, and cytokine and chemokine expression. A group of mice were treated with ABX for 14 days previously and 14 days after TMEV-infection. Once ABX treatment cessation the brains were obtained. (**a**) FACS analysis of leukocytes, macrophages and CD4^+^, CD8^+^ and FoxP3CD25^+^ T cells. (**b**) Representative flow cytometry plots of CD4+ or CD8^+^ cells in gated CD3^+^cells from brains of TMEV or TMEV-ABX mice (n = 6 mice per experimental group). Coronal brain sections (30 μm) from each experimental group were stained for CD4^+^ (**c**) or CD8^+^ (**e**) T cells and counterstained with toluidine blue. Arrows indicates CD4 or CD8 positive T cells. (**d**,**f**) Quantification of number of CD4^+^ or CD8^+^ T cells. Two fields of cerebral cortex per section were analyzed from at least 4 sections for each animal (n = 4 mice per group). (**g**) qRT-PCR analysis of cytokine and chemokine (IL-1β, IL-6, IL-10, TNF-α, IFN-γ, TGF-β, CCL5) expression in brain tissue at 14 dpi. FoxP3 mRNA transcripts were also measured. (**h**) qRT-PCR of VP2 mRNA to analyze the viral load. (TMEV, n = 9; TMEV-ABX, n = 10). The data represent the mean ± SEM and they were analyzed with a Student’s t-test (**a**), non-parametric Kruskal-Wallis test (**d**, **f**) and Mann-Whitney U test (**g**,**h**); ^**^p < 0.01 vs. sham: ^***^p < 0.001 vs sham, ^#^p < 0.05 vs TMEV, ^##^p < 0.01 vs TMEV, ^###^p < 0.001 vs TMEV.

When we inoculated TMEV into the brain we trigger innate immune responses against the virus with infiltration of CD4^+^ and CD8^+^ T cells, as well as B cells and macrophages into the brain^25^. The innate immune response can activate APCs including microglia^26^ and directly contribute to the activation of virus-specific T cells responses which mediate adaptive immunity. It is well known that CD4^+^ and CD8^+^ T cells are important to control TMEV infection. Thus, it was relevant that ABX administration to TMEV-mice reduced the brain proportion of CD4^+^ and CD8^+^ T cells when evaluated by FACS (Fig. 5a,b). Nevertheless, the proportion of brain macrophages (CD45^high^ CD11b^+^) and Treg cells were not significant modified in TMEV-ABX mice (Fig. 5a). We also examined the effect of oral ABX on T cells by analyzing CD4^+^ and CD8^+^ immunostaining in brain sections from sham and TMEV-mice. CD4^+^ and CD8^+^ T cells were present in all TMEV-mice, predominantly located in the perivascular zone, although CD8^+^ T cells were also evident in the CNS parenchyma (Fig. 5c,e) while sham and sham-ABX mice did not show brain CD4^+^ and CD8^+^ infiltrates (Fig. 5c,e). Quantification of T cells revealed that ABX diminished the number of CD4^+^ (Fig. 5d) and CD8^+^ cells (Fig. 5f) without reaching statistical significance, partially consistent with the FACS data. To determine the functionality of these infiltrates and the resident glial cells, particularly microglia, we evaluated the expression of innate immune cytokines in TMEV and TMEV-ABX mice. As expected, and consistent with our earlier data^19^,^25^,^27^, TMEV infection elicited a significant enhancement of cytokines and CCL5 gene expression in the cerebral cortex (data not shown). Interestingly, oral ABX strongly augmented cytokine and chemokine responses to TMEV such as IL-1β, TNF-α, IL-10, IFN-γ, and CCL5 (Fig. 5g). By contrast, oral ABX did not modify IL-6 and TGF-β gene expression (Fig. 5g). We also observed that FoxP3 mRNA transcripts were increased in the cerebral cortex of TMEV-ABX mice (Fig. 5g, p < 0.01). Then, we assessed whether ABX affected TMEV replication and found that at 14 dpi, VP2 mRNA expression was significantly stronger in the brain of TMEV-ABX mice (Fig. 5h). We suggest that the reduced brain CD4^+^ and CD8^+^ cells in TMEV-ABX mice favors virus replication and this drives a strong innate immune response as illustrates the enhanced gene expression of several cytokines and chemokines.

### Oral treatment with ABX affects the immune cell populations in cervical and mesenteric lymph nodes of TMEV-mice on the acute phase

**Figure 6:**
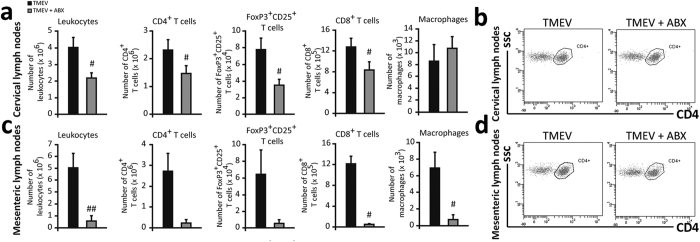
ABX administration diminishes immune cells population in cervical and mesenteric lymph nodes from TMEV-infected mice. After ABX treatment cessation, cervical and mesenteric lymph nodes were obtained. FACS analysis of leukocytes, macrophages and CD4^+^, CD8^+^ and FoxP3CD25^+^ T cells in cervical (**a**) and mesenteric lymph nodes (**b**) from TMEV-infected mice treated with ABX or vehicle. The data represent the mean ± SEM (n = 6 mice per group). Representative FACS plots of gated CD4^+^ T cells in cervical (**c**) or mesenteric lymph (**d**) nodes. Student’s *t*-test: ^#^p < 0.05 vs TMEV, ^##^p < 0.01 vs TMEV.

When FACS was used to compare the CD4^+^, CD8^+^, FoxP3^+^CD25^+^ T cell and macrophage populations, oral ABX significantly reduced the number of leukocytes in cervical lymph nodes compared to TMEV-mice (Fig. 6a). Specifically, a significant reduction in these T cell populations (p < 0.05) was evident in the cervical lymph nodes of the mice without affecting the proportion of macrophages (Fig. 6a). A similar response was also observed in the mesenteric lymph nodes (Fig. 6c) in relation to CD8^+^ T cells (p < 0.05), but in this case, the number of macrophages also diminished significantly (p < 0.05).

### The effect of administering oral ABX during the acute phase of TMEV infection on the disease outcome during the chronic autoimmune phase of TMEV-IDD

**Figure 7:**
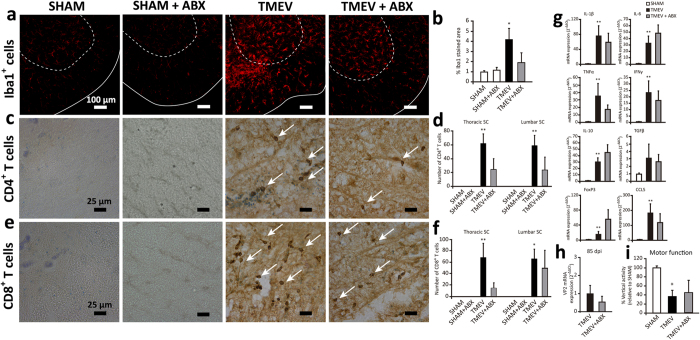
Early ABX treatment does not modify neuroimmune responses to TMEV and disease severity on the chronic phase. A group of mice were treated with ABX for 14 days previously and 14 days after TMEV-infection and were kept alive until chronic phase of TMEV-IDD (85 dpi). (**a**) Representative immunofluorescence of transversal spinal cord section (30 μm) labeled for Iba1 microglia marker. Discontinuous line delimits gray substance while continuous line indicates white substance. (Scale bar 100 μm). Photomicrographs of transversal spinal cord sections immunostained for CD4 (**c**) or CD8 (**e**) T cells. Arrows point to stained cells. Scale bar 25 μm. Quantification of percentage of Iba1 stained area (**b**), number of CD4 (**d**) or CD8 (**f**) T cells. Histological analysis were performed in 6 thoracic-lumbar spinal cord sections per animal from at least 4 animals per group. (**g**) qRT-PCR analysis of cytokine and chemokine (IL-1β, IL-6, IL-10, TNF-α, IFN-γ, TGF-β, CCL5) expression in spinal cord tissue at 85 dpi. FoxP3 mRNA transcripts were also measured (**h**) qRT-PCR of VP2 mRNA to analyze the viral load. (sham, n = 4; TMEV, n = 9; TMEV-ABX, n = 10). (**i**) Motor function was evaluated by the Activity cage for two periods of 5 minutes and vertical activity was registered. The data represent the mean ± SEM analyzed by a non-parametric Kruskal-Wallis test: ^*^p < 0.05 vs sham, ^**^p < 0.01 vs sham.

Finally, we assessed how early ABX administration at the time of TMEV inoculation affected the characteristics of the ongoing chronic phase. This part of the study was performed in spinal cord as pathological abnormalities in TMEV-IDD were prominent in the spinal cord during the chronic phase. While the microglia of spinal cord in sham animals, irrespective of ABX treatment, had the ramified morphology typical of unchallenged cells, activated microglia was present in TMEV-mice at 85 dpi as we have observed in our previous studies^28^. Oral ABX treatment on the acute phase apparently reduced the extension of Iba1 staining on the chronic phase, although without reaching statistical significance (p = 0.059; Fig. 7a,b). Similarly, in the chronic phase of the disease no significant changes in CD4^+^ and CD8^+^ cells were found between TMEV-mice and TMEV-ABX mice (Fig. 7c–f). We did not detect CD4^+^ and CD8^+^ T cell infiltrates in sham and sham-ABX mice. The expression of the canonical Th1 cytokine gene, IFN-γ, the pro-inflammatory cytokines, IL-1β, TNF-α and IL-6, and the anti-inflammatory cytokines, IL-10 and TGF-β, as well as CCL5 and FoxP3 was not significantly affected by the early administration of ABX (Fig. 7g). TMEV-mice showed IL-1β, TNF-α, IL-10, and IFN-γ gene upregulation in the spinal cord at 85 dpi, as seen previously^27^,^29^,^30^. In summary, the administration of ABX on the acute phase of TMEV infection did not significantly modify Iba-1 staining, T cell infiltrates and cytokine expression in the spinal cord on the chronic phase. Likewise, the expression of VP2 mRNA by TMEV-mice was not modified at 85 dpi by early ABX (Fig. 7h). Motor activity is a useful means to accurately assess the neurological deficits in TMEV-infected mice. Although functional disease progression after Theiler’s DA strain inoculation has been evaluated using the accelerated rotarod assay and the activity cage^31^, vertical activity is the parameter most strongly affected in TMEV-mice. Here, disease severity was similar in TMEV-mice irrespective of the early administration of ABX, as reflected by impaired vertical motor activity (Fig. 7i).

## Discussion

The commensal bacteria that inhabit the intestine have an influence on their mammalian hosts that extends well beyond the digestive system. Focusing on CNS, it has been described a symbiotic relationship between microbiota and the CNS, named as the “gut-brain axis”, which is an integrative concept of the bidirectional neurohumoral interplay between CNS and the gastrointestinal system^5^,^32^. Several evidences indicate that the gut microbiome plays a significant role in the progression of demyelinating disease^6^. Moreover, case-control studies in MS patients reveal alterations in gut microbiota composition^33^,^34^. Thus, we set out to analyze whether CNS inflammation by TMEV inoculation could modify the commensal microbiota and how depleting the microbial load affects the immune responses to brain infection with TMEV on the acute phase. The findings we report herein indicate that TMEV infection prompt significant changes in the gut microbiota at different phases of the disease. While *Alloprevotella* population was decreased on the acute phase, *Akkermansia* and *Anaerotruncus* were reduced in presymptomatic stages and *Streptococcus* in the chronic symptomatic time. Both, *Clostridium XIVa* and *Eubacteria* were increased in the presymptomatic and chronic phases, respectively. The abundance of members of *Akkermansia* genus has been suggested as biomarkers for a healthy intestine^35^. Mounting evidence indicates that *Akkermansia* abundance was inversely related to the severity of some diseases like appendicitis^35^, obesity^36^ and children’s autism^37^. Accordingly, we observed a reduction of the relative abundance of *Akkermansia* at 28 dpi in TMEV- infected mice that is a presymptomatic period. Although a recent study^34^ found that *Akkermansia* was increased in MS patients we observed no significant changes on this *Verrucomicrobia* genus during the chronic phase (85 dpi) between TMEV and sham mice. *Akkermansia* species have been reported to be decreased in other autoimmune diseases^38^. Further studies investigating the role of *Akkermansia* in MS are warranted.

It is well known that the gut microbiota population changes overtime. Herein we report that intracranial infection with TMEV modifies the physiological age-related changes observed in the gut microbiota of sham mice by increasing *Clostridium XIVb, Dorea, Eubacterium* or *Oscillibacter* genus between 28 and 85 dpi (see Table 1). Studies in obese mice indicate a negative correlation between *Oscillibacter* genus and transepithelial resistance affecting the intestinal permeability^39^, in agreement with our data in TMEV-mice. As TMEV is a naturally occurring enteric pathogen of the mouse we cannot discard the possibility that the virus exerts direct actions on the gut. Nevertheless, the above possibility is unlikely as clinical disease due to natural Theiler’s virus infection is rare (1 in 1000–10000) and histological lesions in the intestine are not discerned^40^. In fact, we did not detected the presence of virus in the ileum of mice subjected to the intracranial inoculation with TMEV (data not shown).

Oral administration of ABX provoked a loss of the intestinal microbes but recolonization was observed 28 days after TMEV infection with a significant increase in the relative abundance of the *Verrucomicrobia* phylum and a decrease of the *Bacteroidetes* phylum which was related to an inflammatory profile^41^. Accordingly, it is conceivable that these changes were associated with the higher mortality of TMEV-ABX mice. Longitudinal samples of surviving mice indicate that the diversity of the gut microbiota recovers over time and progressively reaches the diversity seen in TMEV-mice at 85 dpi. Therefore, microbiota alterations provoked by oral ABX were temporary and the maintenance of a moderate dysbiosis in TMEV-mice during the autoimmune stage of the disease is consistent with some of the proposed alterations in the gut microbiota of patients with MS^42^.

Several experimental studies have focused on the contribution of the microbiota to MS pathogenesis using different variants of EAE^43^,^44^. GF mice developed a milder EAE clinical course than conventionally colonized ones^13^. Although TMEV-IDD and EAE share pathogenic mechanisms, these two MS models have important differences, then, the consequences of oral ABX on the responses to TMEV must be observed from a perspective different than that applied to EAE.

Macrophages and microglia are the predominant inflammatory cells in active and chronic MS plaques and more importantly, persist in secondary progressive MS as recently has been established^45^. Innate immune responses that occurs after TMEV infection are critical to control the spread of the virus and to limit the damage^46^. Microglia are the first cells to respond to CNS viral infection mediating early control of the pathogen and orchestrating downstream immune reactions. We examined how the depletion of the host’s resident microbes by oral ABX affect microglial responses to TMEV which have been well characterized previously^25^,^27^. Oral ABX particularly affected microglia, as witnessed by their enhanced activation 14 dpi. In fact, more clusters of microglia throughout the cerebral cortex, hippocampus, striatum and brainstem together with MHC class II more strongly expressed appear in TMEV-ABX mice. Activated microglia augments the production of cytokines and chemokines in response to TMEV as showed in previous reports from our group and others^38^,^39^,^41^, likely with the participation of other type of cells. Our finding that TMEV-ABX mice have elevated IL-1β, TNF-α, CCL5 and IL-10 gene expression was consistent with enhanced microglia activation in these mice. The chemokine CCL5, of special interest in the TMEV model^47^, might attract immune cells to the site of virus location while the cytokines can activate the infiltrating cells. The innate immune response activate APCs including microglia^26^ and directly contribute to the activation of virus-specific T cells which mediate the adaptive immune response. Although the treatment of autoimmune conditions has mainly relied on adaptive immunity, recent work points to the relevance of innate immune mechanisms which may contribute to autoantigen generation^48^. In TMEV-IDD upregulated serine proteases like kallikreins was observed in infiltrating monocytes and microglia at sites of CNS inflammation^49^. Microglia of GF mice have been described to show not only morphological alterations but impairment of innate immune responses when challenged by LPS or lymphocytic choriomeningitis virus (LCMV)^50^. Here we do not use GF mice or SPF conditions, but the depletion of microbiota by oral ABX did not modify microglia morphology in sham mice. Furthermore, the responses of microglia to TMEV infection were clearly exacerbated in mice receiving ABX. The depletion of commensal microbial density led here to a defective CD4^+^ and C8^+^ T cell number, coincident with increased expression of the viral VP2 capsid protein, which probably reflects more active viral replication due to a dampened antiviral immune response. Consequently, the exacerbated microglial activation in TMEV-mice with depleted microbiota might be related to increased viral titers. Since TMEV initially infects neurons during the acute phase, spreading thereafter through axonal transport^51^ it is conceivable that the activation of microglia in TMEV-ABX mice in more caudal brain areas reflects more extensive spread of the virus.

From a mechanistic point of view, the role of gut microbiota in viral infection remains relatively unclear. The microbiota may offer protection by preventing viral immunopathology, and its depletion may suppress antiviral immune responses and provoke more active viral replication, as demonstrated after exposure to systemic LCMV or mucosal influenza virus^52^, and consistent with the data presented here. However, certain viruses use the gut microbiota to promote infection, resulting in a more severe clinical course^17^. The variations in the brain infiltrates detected in TMEV-ABX mice included a reduction of CD4^+^ and CD8^+^ T cells, although the total number of macrophages was similar irrespective of ABX. Following brain TMEV infection, CD4^+^ and CD8^+^ T cells can be primed in the periphery and they subsequently infiltrate into the CNS^53^. The dampened cellular immune responses with fewer CD4^+^ and CD8^+^ T cells in the cervical lymphoid nodes from TMEV-ABX mice may in turn contribute to the observation of less infiltrates in the CNS. Our finding that the mesenteric lymph nodes have reduced CD4^+^ CD8^+^ T cells but also fewer macrophages may be related to the elimination of gut bacteria by ABX since the intestinal immune system is tightly regulated to optimize protection against pathogens^54^. The increased intestinal permeability and morphological alterations of the villi in the ileum found in TMEV-mice agree with previous observations in the EAE model^43^ and with the increased number of CD4^+^ T cells we saw in the lamina propria. Nevertheless, oral ABX did not modify the above changes in a significant way. Although in EAE the treatment with ABX diminishes Th17 cells in the Peyer’s patches^6^ we observed a lack of effect in TMEV-IDD. Further studies are necessary to clarify the link between increased permeability associated to brain TMEV inoculation and immunological activities in the gut and related lymphoid organs.

Treg cells play a key role in the maintenance of immunological tolerance and in preventing immunopathologies^55^. However, in viral diseases Tregs can exhibit both beneficial and detrimental effects as their immunosuppressive properties, can cause disease exacerbation or viral persistence^56^. In TMEV-IDD, protective and detrimental roles of Tregs have been described depending on the stage of the disease^57^. Anti-CD25-induced inactivation of Tregs in susceptible SJL/J mice significantly dampens the clinical effects^58^. Our phenotypic analysis of CD4^+^FoxP3^+^CD25^+^ Treg cells following oral ABX failed to identify significant changes in the brain, although increased trascripts of FoxP3 were observed. One striking observation was the enhanced expression of the anti-inflammatory cytokine IL-10 in the brain of TMEV-ABX mice. Although Tregs are an important source of IL-10 during CNS inflammation^59^ macrophages and microglia also expressed IL-10 in the brain of acutely TMEV infected mice^60^ likely contributing to the increase of IL-10 gene expression on the brain of TMEV-ABX mice.

There is evidence that elements of the gut microbiota make diverse and non-redundant contributions to their host’s health. It is known that several microorganisms promote anti-inflammatory networks whereas others induce protective inflammatory responses^41^. Accordingly, it has been suggested that a greater diversity of microbial organisms is better for the host’s health. Here, the depletion of microbiota influences the course of TMEV-IDD in terms of mortality, which is likely to be related to the presence of microbiota of an inflammatory profile at the specific time point of 28 dpi although we cannot demonstrate this in an unequivocal way for obvious reasons. The surviving ABX mice showed similar indices of microbiota diversity than TMEV-mice at chronic stages and they developed similar disease severity with no differences in their CNS viral titers. Since the approach of our study is not mechanistic we cannot assign a direct cause to the association we suggest about microbiota diversity and clinical severity of TMEV disease.

In summary, this study provides novel insights about the potential role of the gut microbiota in TMEV-related neuroimmune responses during the acute phase and their impact on the ongoing disease. Our findings support the bidirectional communication brain-gut axis showing not only that intracranial infection with TMEV induces changes in the gut microbiota but also that depleting microbiota by oral ABX induces changes in the neuroimmune responses to TMEV. Future studies might help to understand the causal direction of our findings from a mechanistic perspective.

## Methods

### Animals and TMEV infection

Four-week-old susceptible female SJL/J mice (Harlan; Barcelona, Spain) were maintained at the Instituto Cajal (CSIC; Madrid, Spain) under controlled house conventional conditions; a 12 h light/dark cycle, at temperature 20 °C (±2 °C) and 40–50% relative humidity, and with *ad libitum* access to food and water. Mice were inoculated in the right brain hemisphere with 2 × 10^6^ plaque forming units (pfu) of the Daniel (DA) strain of TMEV, diluted in 30 μL of DMEM supplemented with 10% of fetal calf serum (FCS), as described previously^61^. Sham mice received 30 μL of the vehicle alone. Each experimental group were housed in different cages and in different isolated racks in the same room with the same food and sterile water, and were manipulated by the same person. There are 4 cages per rack. Each cage contains 6 mice subjected to the same treatment. Therefore the microbiome environment tends to be the same for every mouse. Although coprophagia may lead to more homogeneous microbiota in animals of the same cage that are subjected to the same experimental conditions, not in mice housed in different cages. We never mixed mice from different experimental conditions. All experiments were performed in strict accordance with EU (Directive 2010/63/EU) and Spanish regulations (Decret 53/2013 BOE n° 34 and Comunidad de Madrid: ES 280790000184). The Ethics Committee on Animal Experimentation at the Instituto Cajal (CSIC) approved all the procedures described in this study (protocol number: 2013/03 CEEA-IC).

### Antibiotic treatment

Mice were provided autoclaved drinking water supplemented with ampicillin (1 g/L, Sigma-Aldrich, Madrid, Spain), metronidazole (1 g/L, Sigma-Aldrich, Madrid, Spain), neomycin sulfate (1 g/L, Gibco-Invitrogen, Barcelona, Spain) and vancomycin (0.5 g/L, Sigma-Aldrich Madrid, Spain). ABX treatment started 14 days before TMEV infection and terminated on day 14 post-infection (pi). Animals were sacrificed on day 14 or 85 pi to assess the influence of early ABX treatment on the outcome and severity of the disease.

### Sample collection and massive next generation sequencing

Fecal samples were collected freshly from each mouse on day 14, 28 and 85 pi and stored at −80 °C. After slow thawing at 4 °C over 24 h, DNA was obtained from 0.1 g of feces using the commercial QiaAmp kit (Quiagen; Hilden, Germany). The composition of the bacterial microbiota was determined by next generation sequencing using Ilumina Technology (NGS: performed at the Centro Superior de Investigación en Salud Pública, Valencia University, Spain). Accordingly, 16S rDNA genes were amplified following the Illumina protocol for 16S rDNA gene Metagenomic Sequencing Library Preparation (Part #15044223 Rev. A). The gene-specific sequences used in this protocol target the 16S rDNA gene V3 and V4 region of the 16S rDNA gene. Overhanging Illumina adapter nucleotide sequences were added to the gene-specific sequences and the primers used were as indicated elsewhere^62^. According to standard IUPAC nucleotide nomenclature, the full length primer sequences used to target this region were: 16S rDNA gene Amplicon PCR Forward primer = 5′ TCGTCGGCAGCGTCAGATGTGTATAAGAGACAGCCTACGGGNGGCWGCAG 3′ and 16S rDNA gene Amplicon PCR Reverse primer = 5′ GTCTCGTGGGCTCGGAGATGTGTATAAGAGACAGGACTACHVGGGTATCTAATCC 3′.

We used Microbial Genomic DNA (5 ng/μl in 10 mM Tris, pH 8.5) to initiate the protocol and after 16S rDNA gene amplification, a multiplex step was performed using the Nextera XT Index Kit (FC-131-1096). We ran 1 μl of the PCR product on a Bioanalyzer DNA 1000 chip to verify the size, given an expected size of ~550 bp. After size verification the libraries were sequenced using a 2 × 300 pb paired-end run (MiSeq Reagent kit v3: MS-102-3001) on a MiSeq Sequencer according to the manufacturer’s instructions (Illumina).

Nucleotide filiations were assigned based on the Ribosomal Database Project Samples having removed short sequencing length reads (<200 bp). The quantitative data of the reads were homogenized using their relative percentage from the total reads of each sample to facilitate the comparison between samples.

### Tissue processing and Immunohistochemistry

#### CNS processing and immunohistochemistry

Mice were anesthetized by intraperitoneal injection of pentobarbital (50 mg/kg body weight) and perfused transcardially with saline. The brain (14 dpi) and spinal cord (85 dpi) of each animal was fixed in 4% paraformaldehyde (PFA) diluted in 0.1 M phosphate buffer (PB), cryoprotected in a 30% solution of sucrose in 0.1 M PB and frozen at −80 °C. Free-floating brain and spinal cord cryostat sections (30 μm thick) were washed three times for 10 min with 0.1 M PB and the endogenous peroxidase activity was inhibited in 50% methanol and 1.66% hydrogen peroxide for 90 min. The sections were permeabilized in 0.1 M PB with 0.1% Triton X-100 and blocked for 1 h at room temperature in blocking buffer (0.1 M 1PB plus 0.1% Triton X-100 and 5% animal serum) and then, incubated overnight with the primary antibody against CD4 (1:500) or CD8 (1:1,000; BD Pharmingen, Erembodegem, Belgium). After washing three times for 10 min with 0.1 M PB with 0.1% Triton X-100, the sections were incubated for 1 h with the biotinylated rabbit anti-rat antibody (Vector Laboratories Inc.; Burlingame, CA, USA), the sections were then rinsed for 1 h with the biotin/peroxidase complex (Vector Laboratories Inc.; Burlingame, CA, USA) and antibody binding was detected with the chromogen 3,3′ diaminobenzidine tetrahydrochloride (DAB; Sigma-Aldrich; St. Louis, MO, USA). Finally, the sections were toluidine blue stained, dehydrated, cleared with xylene and coverslipped, confirming the specificity of staining by comparison with stained sections in which the primary antibody was omitted.

For immunofluorescence studies, the sections were rinsed three times for 10 min with 0.1 M PB, permeabilized by 0.1 M PB with 0.1% Triton X-100 and blocked as above and then, incubated overnight with the primary antibody against Iba1 (ionized calcium binding adaptor molecule 1, 1:1,000; Wako Chemical Pure Industry, Osaka, Japan) or MHC-II (1:1,000; AbD Serotec, Oxford, UK). After washing three times for 10 min with 0.1 M PB 0.1% Triton X-100, the sections were incubated for 1 h with an Alexa Fluor-conjugated secondary antibody (1:1,000; Molecular Probes Inc, Eugene, OR, USA). After washing three times for 10 min with 0.1 M PB the sections were mounted with mowiol.

#### Ileum processing and immunohistochemistry

After saline perfusion, the ileum of each animal was dissected out and fixed in 4% paraformaldehyde (PFA) diluted in 0.1 M phosphate buffer (PB), cryoprotected in a 30% solution of sucrose in 0.1 M PB and frozen at −80 °C. Slide-mounted transversal ileum cryostat sections (30 μm thick) were stained for ZO-1 (1:500, Zymed laboratories, CA, USA) and CD4^+^ T cells following protocols described above.

### Intestine permeability

Intestine permeability was studied as previously described^43^. Briefly, mice were gavaged with a marker molecule solution containing 100 μg/g body weight sodium fluorescein (Na-F) (Sigma-Aldrich, MW 376,27 Da) in 0.9% NaCl. After one hour mice were anaesthetized and blood samples were collected. Marker concentrations in blood plasma were measured in 96-microwell plate by spectrophotofluorometer (Fluostar Optima, BMG Labtechnologies, Germany) using a filter setup for 485 nm excitation and 520 nm emission. Standard concentrations of Na-F were used as references.

### Flow cytometry

After 14 dpi, the brain, Peyer’s patches and cervical and mesenteric lymph nodes were dissected and single cell suspensions were obtained as previously described^63^ with minor modifications. Briefly, cell suspensions were passed through a 100 μm filter to remove cell debris and in the case of the lymph nodes incubated for 2 min with 0.8% NH_4_Cl to lyse the erythrocytes. CNS suspensions were diluted in stock isotonic percoll (SIP; GE Healthcare, Buckinghamshire, UK) to a final concentration of 30% SIP, and this suspension was layered on 70% SIP and centrifuged for 30 min at 500 g at 18 °C. The 30–70% interphase was collected, washed and resuspended in FACS buffer (PBS + 0.1% BSA) for immunostaining. Isolated cells were incubated with anti-CD16/CD32 (Affymetrix Inc., Santa Clara, CA, USA) for FcR blockade and then, with antibodies against: APC-Cy7-conjugated anti-CD11b (2.5 μg/ml; BD Pharmingen, Erembodegem, Belgium), PerP-Cy5.5-conjugated anti-CD45 (2.5 μg/ml; BD Pharmingen, Erembodegem, Belgium BD Pharmingen, Erembodegem, Belgium); PE-conjugated anti-CD3 (5 μg/ml; Affymetrix Inc., Santa Clara, CA, USA), APC-Cy7-conjugated anti-CD4 (2.5 μg/ml; BD Pharmingen, Erembodegem, Belgium); PECy7-conjugated anti-CD8 (2.5 μg/ml; BD Pharmingen, Erembodegem, Belgium); APC-conjugated anti-CD25 (0.125 μg/ml: Affymetrix Inc., Santa Clara, CA, USA). Cells were fixed overnight with fixation buffer (Affymetrix Inc., Santa Clara, CA, USA). For FoxP3 detection the cells were suspended in Fixation/Permeabilization buffer overnight and stained with an anti-FoxP3 antibody (0.125 μg/ml; BD Pharmingen, Erembodegem, Belgium). For analysis of intracellular IL-17, cell suspension from Peyer’s patches isolated from TMEV and TMEV + ABX mice at 14 dpi were stimulated as described^43^ and three days later cells were treated with PMA/ionomycin (10 ng/ml; 500 ng/ml, respectively) for 4 h in the presence of monensine (1 μM) and washed. After FcR blockade, cells were fixed with Cytofix/Cytoperm solution and stained with Alexa 700-conjugated anti-IL-17A (10 μg/ml; Biolegends; San Diego, USA). At least 15,000 events were acquired in each experiment on a FACSAria flow cytometer (BD Biosciences; San Diego, CA, USA), excluding duplets from the analysis. The data were analyzed using FACSDiva analysis software (BD Biosciences; San Diego, CA, USA).

### Evaluation of motor function

TMEV-IDD is a model of chronic progressive MS in which virus inoculation is followed by a period of latency until the symptoms and motor deficits appear. Animals were followed for the development and progression of demyelinating disease by evaluating spontaneous motor function. The screening for locomotor activity was performed using an activity cage (Activity Monitor System Omnitech Electronics, Inc.; Colombus, OH, USA) coupled to a Digiscan Analyser to evaluate spontaneous motor activity. Vertical activity is an excellent parameter to evaluate neurological deficits^64^ and these data were obtained as the total number of beam interruptions in the vertical sensor.

### Microscopy and image analysis

Six brain and six spinal cord sections were obtained per animal from at least 4 animals per group and the Iba1 staining was quantified using the Image J software (NIH; Bethesda, MD, USA). Sections were analyzed by immunofluorescence on a Leica TCS SP5 confocal microscope and with a Zeiss Axiocam high-resolution digital color camera for immunohistochemistry. To visualize the rostro caudal spreading of microglia low magnification (10x) images were taken, however to analyze microglial morphology and Iba-1-MHC-II staining we took higher magnification (40x) images. The study of cell morphology were performed by the AnalyzeSkeleton plugin (http://imagejdocu.tudor.lu/) (Fiji software) to collect data on the number of endpoints per cell. More than 500cells/animal were analysed.

### RNA extraction and Real-Time Polymerase Chain Reaction

Total RNA was isolated from the prefrontal cortex for TMEV-IDD acute phase experiments and from cervical spinal cord tissue for the chronic autoimmune phase using RNeasy mini columns (Qiagen; Manchester, UK), removing genomic DNA contamination by DNase I digestion (DNase I; Sigma-Aldrich Química SA, Madrid, Spain). Total RNA (1 μg) was reverse transcribed into cDNA using poly-dT primers and a reverse transcription kit (Promega Biotech Ibérica S.L., Madrid, Spain) and this cDNA was used as the template for real-time PCR performed using SYBR^®^ and the oligonucleotide primer sequences indicated in Table 2 (Applied Biosystems; Warrington, UK). PCRs were initiated with the incubation at 50 °C for 2 min and 95 °C for 10 min, and the PCR amplification was performed over 40 cycles of 95 °C for 15 s and 60 °C for 1 min. The cDNA samples were analyzed in triplicate on an Applied Biosystems PRISM 7500 Sequence detection system, normalizing the mRNA expression to the RPS29 gene in each sample and quantifying gene expression by the 2^−ΔΔCt^ method. The results are expressed relative to the TMEV or sham mice for each time point.

### Statistical analysis

For the gut microbiota composition statistics were performed with the R Statistics software- (http://www.R-project.org/); the pipeline core run in R Rstatistics language (R Core Team, 2012). All calculations and statistics were carried out within this environment using the following packages: lattice-graphics for R^65^; knitr, knitcitations, markown- report and reference environment^66^,^67^,^68^; Markdown- Markdown rendering for R. R package (version 0.6.4); and Bioconductor packages for genomics data- Biostrings. ANOVA with repeated measures was applied to identify the significant differences in the phyla or genera composition between the different times of sampling, whereas the post-hoc Bonferroni correction was used to compare the different sets in each time. Both analyses were performed on the SPSS 22 software (IBM Corporation; USA). The Simpson, Shannon and Chao1 diversity indices were performed using Past 3.07 software.

The SPSS 22 software also was used for the rest of statistical analysis, applying parametric Student’s t-test and non-parametric Kruskal-Wallis test or Mann-Whitney U tests. All the data are presented as the mean ± standard error of mean (SEM), unless otherwise indicated. A value of p < 0.05 was considered statistically significant.

## Additional Information

**How to cite this article:** Carrillo-Salinas, F. J. *et al*. Gut dysbiosis and neuroimmune responses to brain infection with Theiler’s murine encephalomyelitis virus. *Sci. Rep.* **7**, 44377; doi: 10.1038/srep44377 (2017).

**Publisher's note:** Springer Nature remains neutral with regard to jurisdictional claims in published maps and institutional affiliations.

## Figures and Tables

**Table 1:** Summary of temporal relative abundance changes of sham and TMEV-mice microbiota.

	Decrease	Increase
	(14–28 dpi)	(28–85 dpi)
*Anaerotruncus*	sham (p = 0.109)	sham (p = 0.486)
	TMEV (**p **=** 0**.**001**)*	TMEV (p = 0.243)
*Clostridium XIVb*	sham (**p **=** 0**.**008**)*	sham (p = 0.486)
	TMEV (**p **=** 0**.**0001**)*	TMEV (**p **=** 0**.**008**)*
*Dorea*	sham (p = 0.109)	sham (p = 0.114)
	TMEV (**p **=** 0**.**002**)*	TMEV (**p **=** 0**.**035**)*
*Eubacterium*	sham (**p **=** 0**.**028**)*	sham (p = 0.114)
	TMEV (**p **=** 0**.**006**)*	TMEV (**p **=** 0**.**0001**)*
*Lachnospiracea_incertae_sedis*	sham (p = 0.383)	sham (p = 1)
	TMEV (**p **=** 0**.**0004**)*	TMEV (p = 0.421)
*Oscillibacter*	sham (p = 0.109)	sham (p = 1)
	TMEV (**p **=** 0**.**0001**)*	TMEV (**p **=** 0**.**006**)*
*Pseudoflavonifractor*	sham (p = 0.299)	sham (p = 0.92)
	TMEV (**p **=** 0**.**0001**)*	TMEV (p = 0.216)
	**Increase**	**Decrease**
	(**14–28 dpi**)	(**28–85 dpi**)
*Akkermasia*	sham (**p **=** 0**.**004**)*	sham (**p **=** 0**.**029**)*
	TMEV (**p **=** 0**.**0001**)*	TMEV (**p **=** 0**.**022**)*
*Enterorhabdus*	sham (p = 0.882)	sham (p = 0.144)
	TMEV (p = 0.159)	TMEV (**p **=** 0**.**002**)*

^*^The changes that reach statistical significance are showed in bold words. Sham (n = 4); TMEV (n = 9).

**Table 2:** The mouse primer sequences used in quantitative Polymerase Chain Reactions.

Genes	Forward	Reverse
VP2 (TMEV)	5′-TGG TCG ACT CTG TGG TTA CG-3′	5′-GCC GGT CTT GCA AAG ATA GT-3′
IL-1β	5′-TGG TGT GTG ACG TTC CCA TT-3′	5′-TCC ATT GAG GTG GAG AGC TTT C-3′
IL-6	5′-TCC AGA AAC CGC TAT GAA GTT C-3′	5′-CAC CAG CAT CAG TCC CAA GA-3′
IL-10	5′-TGA ATT CCC TGG GTG AGA AGC TGA-3′	5′-TGG CCT TGT AGA CAC CTT GGT CTT-3′
TNF-α	5′-AGA GGC ACT CCC CCA AAA GA-3′	5′-CGA TCA CCC CGA AGT TCA GT-3′
IFN-γ	5′-GGC CAT CAG CAA CAA CAT AAG CGT-3′	5′-TGG GTT GTT GAC CTC AAA CTT GGC-3′
TGF-β1	5′-CCA GCC GCG GGA CTC T-3′	5′-TTC CGT TTC ACC AGC TCC AT-3′
FoxP3	5′-GCC CAC ACC TCT TCT TCC TTG AA-3′	5′-TGA AGT GTG GTC TGT CCT GGA GAA-3′
RANTES	5′-TCG TGC CCA CGT CAA GGA GTA TTT-3′	5′-TCT TCT CTG GGT TGG CAC ACA CTT-3′
RPS29	5′-GCC GCG TCT GCT CCA A-3′	5′-ACA TGT TCA GCC CGT ATT TGC-3′
